# Influence of Mold Design and Molding Conditions on the Optical Properties of Contact Lenses

**DOI:** 10.3390/s26103007

**Published:** 2026-05-10

**Authors:** Kazumasa Tatsumi, Kentaro Saeki, Shin Kubota, Yoshikatsu Kaneda, Kenji Uno, Kazuhiko Ohnuma, Tatsuo Shiina

**Affiliations:** 1 SEED Co., Ltd., 2-40-2 Hongo, Bunkyo-ku, Tokyo 113-8402, Japan; k_saeki@seed.co.jp (K.S.);; 2 Graduate School of Science and Engineering, Chiba University, 1-33 Yayoi-cho, Inage-ku, Chiba 263-8522, Chiba, Japan; 3 Laboratorio de Lente Verde, 98-1 Nozomino, Sodegaura 299-0251, Chiba, Japan

**Keywords:** contact lenses, resin mold, injection molding

## Abstract

Injection molding is essential for mass-producing soft contact lenses, yet molding-induced deformation remains a decisive factor for optical quality. This study systematically evaluated the impact of resin mold design factors (optical zone (OZ) radius of curvature and resin mold thickness) and injection molding parameters (holding pressure and injection speed) on the properties of a dry-state lens (dry lens) using an L18 orthogonal array. The results demonstrated that optimizing the resin mold thickness to 0.9 mm reduced astigmatism by approximately 95%, while high holding pressure and low injection speed improved structural stability. Notably, the findings suggest that the refractive power of the dry lens is more strongly governed by macro-level curvature fluctuations and internal stress distributions arising from the resin mold thickness and shape than by the wavefront aberrations of the resin mold itself. Designs with a smaller radius of curvature (R = 6.5 mm) exhibited substantial power deviations of up to +2.8 D, whereas deviations remained within ±0.2 D for designs with a larger radius of curvature (R = 8.5 mm). For high-precision lens manufacturing, it is indispensable to incorporate a resin mold design that accounts for deformations induced during molding. This study provides comprehensive guidelines for achieving high-quality products by detailing the relationship between injection molding and design.

## 1. Introduction

Since the invention of the vertical celluloid molding machine by John W. Hyatt in 1872, injection molding has served as the cornerstone of the plastics industry [1]. Driven by advancements in control systems and material science during the latter half of the 20th century, the technology has transitioned from a mere means of mass production into a high-precision manufacturing discipline capable of micrometer-scale accuracy [2]. Notably, in recent years, sophisticated injection mechanisms, such as the V-LINE^®^ system, have emerged, decoupling plasticization from injection to ensure stable melt pressure. This approach has demonstrated superior repeatability compared to conventional reciprocating screw systems, even in demanding applications like Metal Injection Molding (MIM), where extreme dimensional tolerances are mandatory [3]. Consequently, injection molding has solidified its status as an indispensable technology for the fabrication of precision optical components and medical devices.

A prominent application of this precision injection molding technology is the mass production of soft contact lenses (CLs). Currently, the double-sided molding process is the predominant method for contact lens manufacturing. In this process, a pair of precision-designed and injection-molded resin molds (concave and convex) is prepared. The CL monomer is dispensed into the concave mold, and the convex mold is then fitted to it to define the CL shape. Subsequently, the monomer is polymerized to form a solid. Upon removal of the resin molds, a dry-state lens (dry lens) is obtained. Finally, the CL is completed by hydrating the dry lens with water [4]. In this workflow, the optical performance of the final lens hinges fundamentally on the geometric integrity of these intermediate resin molds. However, resin molds are highly susceptible to warpage and dimensional deviations during post-molding cooling, driven by resin shrinkage and residual stresses. Prior research has demonstrated that subtle fluctuations in holding pressure and mold temperature profoundly influence the anisotropic shrinkage and resulting shape accuracy of spherical lenses [5]. Consequently, to ensure stable, high-quality lens production, precise control over shrinkage behavior through optimized molding parameters—beyond mere surface fidelity and replicability—represents a critical technical imperative.

In prior studies, the present authors established a measurement framework utilizing a Shack-Hartmann reflective wavefront sensor and optical coherence tomography (OCT) for the non-contact, high-precision evaluation of the optical properties of resin molds and the dry lens [6,7]. Notably, while wavefront analysis permits evaluation with sub-nanometer precision, our previous findings identified instances in which wavefront aberrations derived solely from the resin mold failed to fully account for refractive power deviations in the final CL. This discrepancy suggests a complex interaction between nonuniform shrinkage induced by injection-molding conditions and geometric deformation driven by heat and pressure during polymerization.

In optical elements, excessive aberrations and geometric deviations not only impair imaging performance but also constitute critical non-conformities in manufacturing quality control. Notably, it has been reported that wavefront aberrations—mathematically characterized by Zernike polynomials—can exert markedly different effects on visual quality even when the root-mean-square (RMS) error remains constant [8]. Thus, minimizing specific aberration modes is vital to preserving vision quality. Furthermore, in injection-molded high-precision aspheric lenses, geometric deviations arising during the molding cycle have been shown to correlate directly with a decrease in optical fidelity [9]. In replication workflows such as CL manufacturing, the ability to predict and govern the shrinkage and expansion of the resin across the mold-to-resin-to-lens sequence is paramount. Given that resin exhibits thickness-dependent differential shrinkage during the melt-flow and cooling phases, the resin mold thickness design transcends its role as a mere structural support; it serves as a decisive control factor in mitigating anisotropic molding shrinkage and maintaining the target curvature.

In addition to conventional optical systems, metasurface-based lenses have recently attracted significant attention due to their compact form factor and advanced wavefront control capabilities [10]. In particular, tunable metasurfaces with adjustable focal lengths enable dynamic focusing without mechanical movement, making them promising for integration into compact optical systems. Such functionality may be advantageous for curvature radius measurements of contact lens molds, as it provides increased flexibility in focal adjustment. However, challenges such as fabrication complexity remain. Therefore, although further technological development is required for practical implementation, the fiber-based configuration employed in this study provides a promising platform for integration with these advanced optical technologies.

The objective of this study is to systematically elucidate how resin mold thickness design and injection molding parameters impact CL characteristics. Specifically, focusing on resin mold thickness, we quantitatively evaluate the influence of these design variables on post-molding geometric deformation by integrating wavefront aberration analysis via a reflective wavefront sensor with three-dimensional thickness distribution mapping via OCT. Furthermore, by deriving optimization guidelines for resin mold design that account for shrinkage and expansion phenomena, this research aims to establish a robust molding process framework. This framework is intended to suppress micro-scale morphological deviations and ensure high fidelity to target optical design specifications.

## 2. Materials

**Figure 1:**
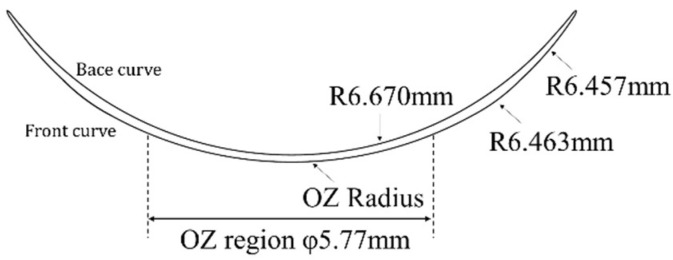
The dry lens design specifications.

**Figure 2:**
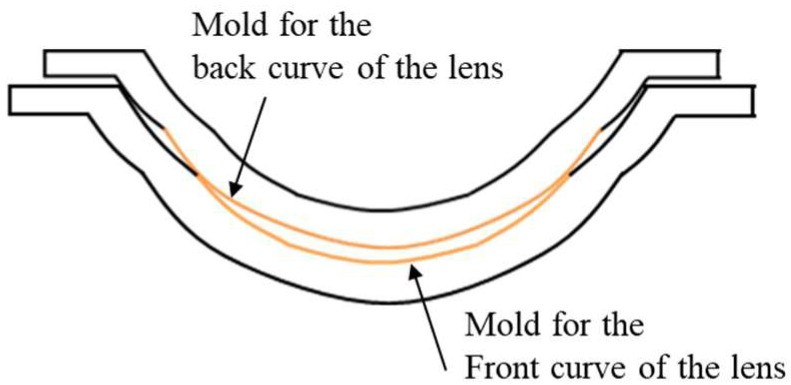
The resin mold configuration used in the manufacturing process.

The evaluation targets in this research consist of the injection-molded resin molds and the resulting CL in their dry state (dry lens). This focus on the dry lens was strategically chosen to evaluate how resin mold design and injection molding parameters dictate lens morphology, while isolating these effects from confounding variables such as hydration-induced swelling. By analyzing the dry lens, it is possible to directly characterize the fidelity of shape transfer from the mold to the dry lens, as well as the geometric deviations inherent to the molding process. Figure 1 illustrates the dry lens design specifications, and Figure 2 shows the resin mold configuration used in the manufacturing process.

A dry lens is fabricated by dispensing liquid monomer between a pair of resin molds and then polymerizing it. In general, the OZ—the region responsible for refractive correction—is governed by the geometry of the Front Curve (FC) mold. Accordingly, this study designates the FC resin mold as the primary subject of evaluation, employing the OZ radius of curvature design and resin mold thickness as variable parameters. In contrast, the design specifications for the peripheral region were kept constant across all samples, and the geometry of the Base Curve (BC) mold was unified throughout all experimental conditions to ensure consistency.

Optical characterization of the resin molds and CLs was conducted using measurement systems tailored to their respective material properties. For the evaluation of the resin molds—which function as reflectors due to their opaque substrates—a Shack-Hartmann reflective wavefront sensor was employed to measure wavefront aberrations and the radius of curvature. Simultaneously, the resin mold thickness was characterized via Optical Coherence Tomography (OCT). In contrast, evaluating dry lenses poses a significant challenge for reflective systems because of their extreme thinness and high transparency. To address this, a transmission wavefront sensor (Lambda-X s.a., Nivelles, Belgium, NIMO TR1504; NIMO) was utilized to quantify refractive power and transmitted wavefront aberrations [11,12].

Furthermore, three-dimensional geometric assessments via OCT were performed to corroborate the findings obtained from both NIMO and the reflective wavefront sensors. By integrating these diverse metrological approaches, we quantified the correlation between resin mold design variations and the resulting optical performance of the dry lens. Detailed diagrams of these techniques are provided in Section 3.

To efficiently achieve both precise refinement of the resin mold’s geometric profile and the minimization of subtle morphological deviations, an orthogonal array-based experimental design was formulated. This methodology offers the advantage of evaluating the main effects of each factor independently—under the assumption that interactions between control factors are sufficiently small—thereby enabling a comprehensive understanding of each factor’s impact with a minimal number of trials [13]. The control factors are detailed in Table 1. As design variables, “OZ radius of curvature” and “resin mold thickness” were selected. For the radius of curvature, 6.5 mm and 8.5 mm were chosen, representing the lower and upper limits in practical CL design. Regarding resin mold thickness, variations of ±0.1 mm relative to the design value (±0.8 mm) (0.7 and 0.9 mm) were introduced to evaluate the impact of resin mold thickness differences on design fidelity and aberration generation. Furthermore, “holding pressure” and “injection speed” were selected as molding parameters due to their documented influence on dimensional stability [14,15]. Table 2 presents the assignment of each level to the L18 orthogonal array. As a mixed-level orthogonal array, the L18 matrix allows evaluation of the effects of individual control factors without being confounded by interactions between specific pairs of factors [16]. By employing this framework in a system where design variables and molding processes coexist, we aimed to identify the dominant factors contributing to shape retention and to establish optimal process guidelines.

## 3. Methods

Under the experimental conditions outlined in Table 1 and Table 2, the evaluation focused on injection-molded resin molds and the resulting polymerized, dry lens. Thickness measurements for both the resin molds and the dry lens were performed using non-contact Optical Coherence Tomography (OCT), which was also used for the morphological assessment of the lenses [17]. Among the various OCT modalities that resolve depth-resolved structures via low-coherence interferometry, this study employed Time-Domain OCT (TD-OCT). This method acquires interference signals by mechanically scanning the reference optical path, a mechanism that enables a long working distance and an extensive measurement range in the optical probe design [18].

**Figure 3:**
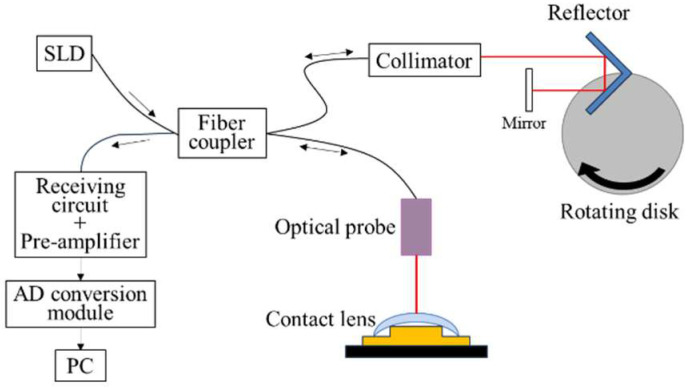
TD-OCT system used in this study.

**Figure 4:**
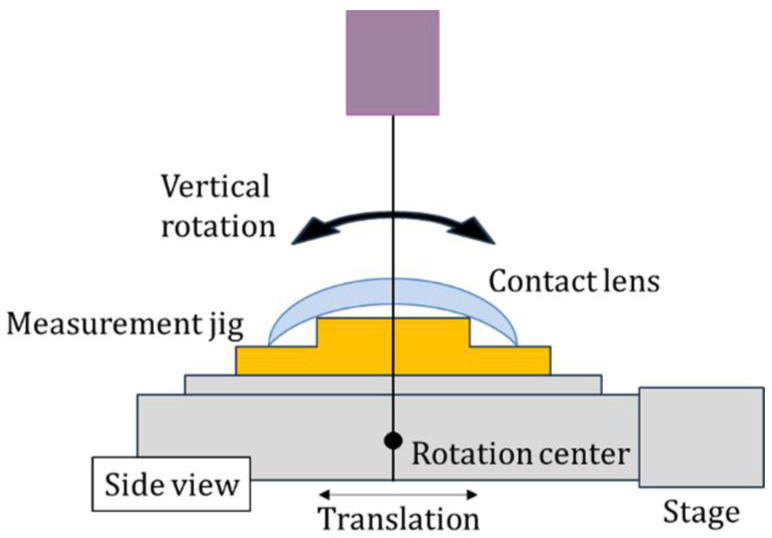
Sample stage overview on OCT measurement.

A significant advantage of TD-OCT is that the measurement probe configuration can be established independently of other performance parameters, such as resolution, scanning speed, and measurement range. Moreover, because the interference signals are linearly amplified, the system exhibits high signal linearity, ensuring superior measurement accuracy [19]. A schematic representation of this measurement framework is provided in Figure 3 and Figure 4.

During the metrological process, a normal-incidence configuration relative to both the front and base dry lens surfaces was maintained by precise control of the measurement stage. The stage incorporates two degrees of freedom—translational motion and vertical-axis rotation—enabling identification of the orthogonal entry point by monitoring the intensity ratio of interference signals from both surfaces. In the OCT framework, the time delay between the two discrete interference signals serves as the basis for calculating local thickness.

The raw interference data were first mapped to spatial distance information based on the reflector’s rotational velocity, then transformed into Cartesian coordinates for both surfaces using the stage’s displacement and angular orientation. To accurately calculate the posterior coordinates, the group refractive index of the CL material was compensated. The radius of curvature was subsequently estimated by applying a least-squares circular fit to the resulting coordinate sets. A key advantage of this methodology is its ability to perform simultaneous, co-axial assessment of both surfaces in a fixed orientation, making it highly effective not only for thickness mapping but also for characterizing inter-surface misalignment.

The dioptric power of the dry lens was calculated from the radii of curvature derived from geometric measurements. Furthermore, the thickness measurements facilitated the acquisition of all essential parameters—specifically center thickness and surface profiles—required for the comprehensive determination of refractive power.

**Figure 5:**
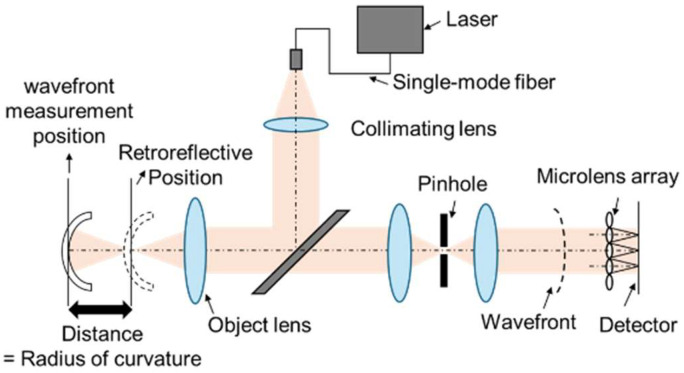
Principle of the reflected wavefront sensor.

The configuration of the reflective wavefront sensor developed for resin mold characterization is illustrated in Figure 5. A fiber-pigtail light source was utilized, and the divergent output from the fiber was transformed into a collimated beam via a collimating lens. To facilitate the measurement of resin molds possessing inherent curvature, a condensing lens was positioned immediately proximally to the sample. Light reflected from the mold surface passes through a microlens array before reaching the imaging sensor. By integrating a pinhole aperture immediately preceding the sensor, the sample surface and the microlens array’s posterior focal plane are rendered optically conjugate, thereby ensuring high-fidelity measurement of wavefront aberrations.

The local gradients of the partitioned wavefront are detected as focal-spot displacements. These gradients are reconstructed using Zernike polynomials—a set of orthogonal basis functions defined over a unit disk—to quantify the wavefront profile. Each term of the Zernike polynomials has an independent wavefront shape and corresponds to an aberration. Using Zernike polynomials $Z_{n}^{m}(X,Y)$, the incident equiphase surface W(X,Y) is expressed as in Equation (1):

(1) $$W\left(X,Y\right)=\sum_{i=0}^{n}\sum_{j=0}^{i}c_{i}^{2j-1}Z_{i}^{2j-1}\left(X,Y\right)$$

Defining the focal spot displacements as $\left(∆x,∆y\right)$ and the focal length of the microlens array as f, the relationship governing the local wavefront slope is expressed as shown in Equation (2):

(2) $$\frac{\partial W(X,Y)}{\partial X}=\frac{∆x}{f}\;\;\;\;\frac{\partial W(X,Y)}{\partial Y}=\frac{∆y}{f}$$

The wavefront can be reconstructed by substituting Equation (1) into Equation (2) to determine the Zernike coefficients. While 36 Zernike parameters are fundamentally defined, this study identified and discussed the notable coefficients that exhibited significant sensitivity to the experimental variables [7]. Furthermore, as shown in Figure 5, the radius of curvature was defined as the distance between the wavefront measurement position and the retroreflection position.

By integrating the thickness data obtained via OCT with wavefront and radius of curvature measurements from the sensor, we quantitatively evaluated morphological variations as a function of both the resin mold design and injection molding conditions.

Conversely, since dry lenses are characterized by their extreme thinness and transparency, their refractive power was quantified using the NIMO system. This was complemented by geometric assessments via OCT [6], from which the dioptric power was independently determined from the measured radius of curvature. By integrating these three distinct datasets—NIMO-derived power, OCT-derived power, and theoretical power calculated from reflective wavefront measurements of the resin molds—we conducted a comprehensive comparative analysis of shape-transfer characteristics. The refractive power “D” was determined based on the focal length “f”, the refractive index of the dry lens material “n”, the radii of curvature of the front and back surfaces “$R_{FC}$” and “$R_{BC}$”, and the center thickness of the dry lens “t”, using the following thick-lens equation:

(3) $$D=\frac{1}{f}=\left(n-1\right)\left(\frac{1}{R_{FC}}-\frac{1}{R_{BC}}\right)+\frac{{\left(n-1\right)}^{2}t}{nR_{FC}R_{BC}}$$

## 4. Results

### 4.1. Wavefront Evaluation of Resin Molds Using Zernike Coefficients

**Figure 6:**
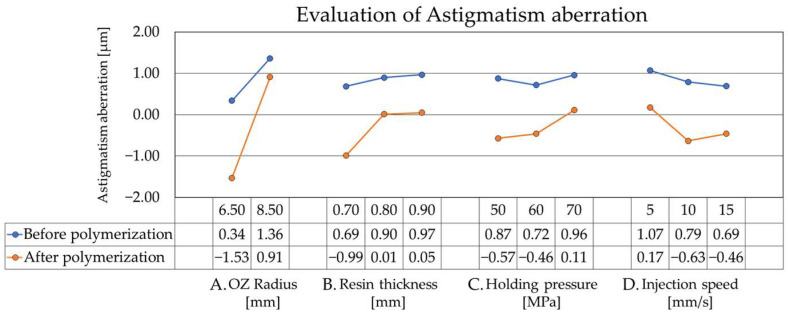
The results for astigmatism aberration.

The results of wavefront aberration measurements using the reflective wavefront sensor are presented. Drawing on previous findings, this evaluation focuses on astigmatism and coma, as these aberrations are known to fluctuate significantly with design parameters and the polymerization process [7]. Other aberrations, such as trefoil and spherical aberration, were also evaluated, while they remained significantly smaller and showed negligible variation compared to astigmatism and coma. Therefore, this paper focuses on these two major aberrations. Figure 6 shows the results for astigmatism, with the horizontal axis representing the four selected parameters—OZ radius of curvature, resin mold thickness, holding pressure, and injection speed—and the vertical axis representing the magnitude of the astigmatism coefficient. The solid lines between data points are provided as visual guides to indicate trends and do not imply a physical model or accurate interpolation of the optical properties between measured conditions. By comparing the states before and after polymerization, several key trends were identified. As the OZ radius of curvature design increased, the astigmatism Zernike coefficient also increased. Furthermore, the thermal history during polymerization caused the Zernike coefficients to shift in the negative direction, a trend consistent with previously reported design and polymerization-dependent aberration changes [7]. Significant differences in aberration were also observed with respect to resin mold thickness. Specifically, the magnitude of change during polymerization was highly sensitive to this parameter; thinner resin molds exhibited substantially larger fluctuations. Regarding the holding pressure conditions, distinct differences emerged post-polymerization. Higher holding pressure correlated with a smaller change magnitude, suggesting enhanced stability. Similarly, slower injection speed contributed to minimized fluctuations before and after polymerization, further promoting stability. In summary, for astigmatism suppression, optimal results were achieved with a thicker resin mold design, high holding pressure, and a slow injection speed.

**Figure 7:**
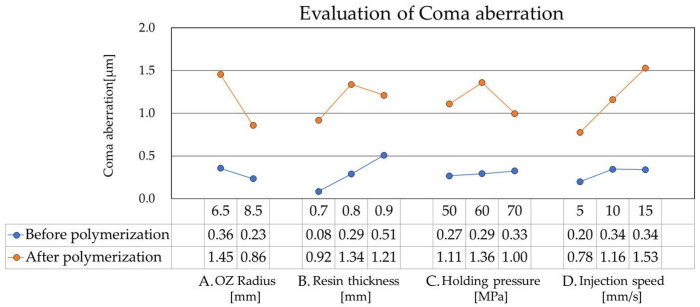
The results for coma aberration.

Next, Figure 7 presents the results for Coma aberration, using the same parameters on the horizontal axis as in Figure 6, with the Coma magnitude on the vertical axis. The observed variations in aberration relative to the OZ radius of curvature were consistent with previous findings [7]. Notably, the trend for resin mold thickness was distinct from that of astigmatism: thinner mold designs tended to yield lower Coma magnitudes. Conversely, the influences of holding pressure and injection speed aligned with the trends observed for astigmatism. While higher holding pressure initially resulted in larger Coma values prior to polymerization, the subsequent magnitude of change was significantly reduced at higher pressures, leading to a reversal in relative aberration values post-polymerization. Regarding injection speed, slower settings consistently produced more favorable outcomes. In conclusion, to minimize Coma aberration and achieve optimal mold stability, the data suggests a strategy of using a thinner resin mold thickness, combined with high holding pressure and slow injection speed. Notably, these conditions did not increase other aberrations, ensuring a balanced improvement in overall wavefront quality.

### 4.2. Evaluation of the Dry Lens Fabricated Under Varying Conditions

The dry lenses fabricated using the resin molds evaluated in Section 4.1 were characterized. This evaluation compared the following three methodologies: (1) measured refractive power of the dry lens using NIMO, (2) calculated equivalent power based on the OZ radius of curvature of the resin molds measured by the wavefront sensor, and (3) calculated equivalent power based on the OZ radius of curvature of the dry lens measured by OCT. For methods (2) and (3), the values were derived using Equation (3). The calculation utilized a known refractive index and the dry lens thickness values obtained via OCT.

**Figure 8:**
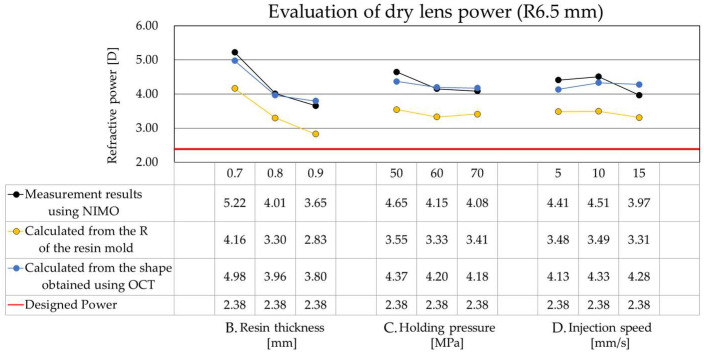
The evaluation results of the dry lens power with an OZ radius of curvature of 6.5 mm.

Figure 8 shows the evaluation results of the dry lens power using a resin mold with a designed OZ radius of curvature of 6.5 mm. The horizontal axis shows three parameters—resin mold thickness, holding pressure, and injection speed—and the vertical axis indicates the dry lens power, with data from the three methods plotted together. The black line represents the dry lens measurement results obtained using NIMO. The orange line indicates the dry lens power calculated from the radius of curvature of the resin mold measured by a wavefront sensor. The blue line shows the power calculated from the dry lens’s radius of curvature, obtained from OCT measurements. The red line represents the design value of the dry lens power. Among the parameters, resin mold thickness had the greatest impact on dry lens power, while holding pressure and injection speed had negligible effects. The direct measurements of the dry lenses using NIMO and OCT (black and blue lines) were generally in agreement. However, discrepancies were observed between the dry lens power based on resin mold measurements (orange line) and the direct measurements from NIMO and OCT (black and blue lines). Furthermore, a deviation from the design value (red line) was also noted. The reasons for these differences in measurement results will be discussed in Section 5.

**Figure 9:**
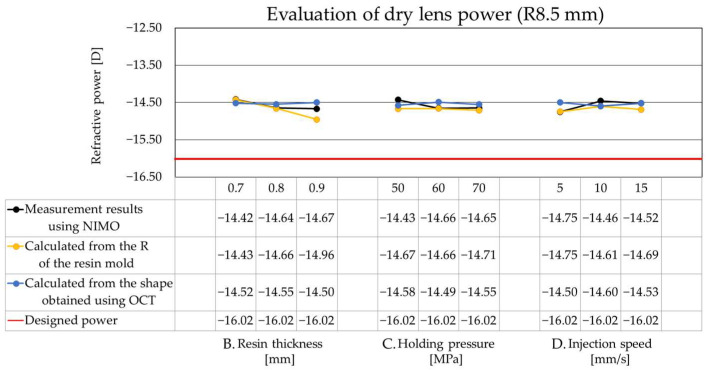
The evaluation results of the dry lens power with an OZ radius of curvature of 8.5 mm.

Next, Figure 9 shows the evaluation results of the dry lens power using a resin mold with an OZ radius of curvature of 8.5 mm. The data were plotted and evaluated in the same format as in Figure 8. With this design, a consistent refractive power was obtained across all conditions. There was an excellent agreement between the dry lens power calculated from the resin mold measurements (orange line) and the direct measurements of the dry lenses obtained via NIMO and OCT (black and blue lines). These results suggest that for the 8.5 mm design, a stable refractive power can be achieved independently of the resin mold thickness or injection molding conditions, indicating that the geometric profile of the resin mold is accurately transferred to the dry lens. However, a constant deviation from the design value (red line) was still observed. The following section analyzes the generation of aberrations and their impact on dry lens power under different design and injection molding conditions, to determine the optimal molding parameters. Additionally, we will investigate the factors contributing to the differing power trends between the 6.5 mm and 8.5 mm designs and discuss the resulting feedback for the CL manufacturing process.

## 5. Discussion

In this study, the effects of resin mold design (OZ radius of curvature and resin mold thickness) and injection molding conditions (holding pressure and injection speed) on the dimensional stability of resin molds and the optical characteristics of the dry lens were systematically evaluated based on orthogonal array experiments. This comprehensive evaluation integrated several methodologies: wavefront aberration analysis of the resin molds using a reflective wavefront sensor, thickness and shape measurements via OCT, and refractive power measurements of the dry lens using NIMO. Through this multi-faceted approach, we investigated the shape-transfer behavior from the resin mold to the final dry lens.

### 5.1. Influence of Molding Conditions on Wavefront Aberration in Resin Molds

Wavefront analysis reveals that astigmatism and coma are highly dependent on the resin mold thickness and injection molding conditions. Specifically, under thinner resin mold thickness conditions, steep temperature gradients during cooling and polymerization likely induce asymmetric shrinkage and residual stress. In this study, astigmatism exhibited extremely sensitive behavior; by increasing the resin mold thickness from 0.7 mm to 0.9 mm, the aberration value decreased from 0.99 µm to less than 0.05 µm—a substantial reduction of approximately 95%. Consequently, low-order Zernike coefficients, such as astigmatism and coma, tended to fluctuate significantly before and after polymerization. This behavior aligns with previous reports stating that asymmetric shrinkage in injection-molded optical lenses manifests as low-order aberrations [20,21].

Furthermore, a thicker resin mold increased the mold’s structural rigidity and promoted a uniform temperature distribution during cooling, thereby reducing wavefront aberration fluctuations throughout the polymerization process. The effectiveness of controlling temperature history via resin mold thickness to reduce residual stress and morphological variation is consistent with recent findings in precision injection molding [22,23].

Regarding holding pressure conditions, higher pressure was associated with lower aberration fluctuations and improved shape reproducibility. By setting a high holding pressure of 70 MPa, we successfully decreased both astigmatism and coma. This is attributed to the stabilized resin filling and suppressed volumetric shrinkage during cooling. Such stabilization effects of holding pressure are recognized as dominant factors in reducing warpage and optical aberrations in molded lenses [24]. However, a slight increase in Coma values was observed at excessively high holding pressure, suggesting that over-packing may increase internal residual stress, which manifests as localized asymmetric distortion [21].

In terms of injection speed, slower conditions reduced the change in aberration during polymerization, yielding a more stable wavefront profile. Reducing the injection speed decreased the coma aberration value by approximately 50% (from 1.53 µm to 0.78 µm). This improvement is likely due to the suppression of molecular orientation and shear stress during resin flow, which decreased the generation of internal residual stress [13,20]. Based on these wavefront aberration results, we conclude that combining a thicker resin mold, a high holding pressure, and a slow injection speed are optimal conditions for enhancing the dimensional stability of the resin mold.

### 5.2. Relationship Between Resin Mold Shape and Dry Lens Power

In the evaluation of dry lenses fabricated using the resin molds, a significant discrepancy was observed for the 6.5 mm OZ radius of curvature design (Design power: +2.38 D). This deviation appeared between the power calculated from the wavefront sensor measurements, the actual dry lens power measured by NIMO and OCT, and the theoretical design power. Specifically, the measured dry lens power consistently exceeded the design power (+2.38 D) across all molding conditions. For the 0.7 mm thickness condition, an extremely large error occurred with a power of 5.22 D (a deviation of +2.84 D). However, increasing the thickness to 0.9 mm decreased deviation, resulting in a power of 3.65 D (+1.57 D deviation). The +2.84 D deviation from the design value observed in this study exceeds the acceptable tolerance for products (±0.25 D) and therefore represents a significant error. This deviation is attributed to macroscopic shape changes in the resin mold caused by the molding and thermal processes. In contrast, the aberrations generated in the resin mold are influenced by the flow and polymerization shrinkage that occur as the monomer transitions from a liquid to a solid state, resulting in the relaxation and averaging of fine surface irregularities and higher-order aberration components; thus, they are not necessarily directly transferred. In fact, the RMS values evaluating the aberration variation are in the sub-micron range, indicating minimal deviation. Therefore, the final lens power can be compensated through design feedback, and it can be concluded that controlling the macroscopic shape is more critical than mitigating aberrations.

In contrast, for the 8.5 mm OZ radius of curvature design (Design power: −16.02 D), the measured values remained within a stable range of −14.4 D to −14.7 D across molding conditions, showing excellent agreement with the design, calculated, and measured values. These results indicate that for designs with a smaller OZ radius of curvature (6.5 mm), minute geometric errors arising from the resin mold thickness or molding conditions are amplified as significant errors in dry lens power. As shown in Equation (3), since optical power is proportional to the reciprocal of the radius of curvature, even a slight curvature deviation increases the power error more prominently in high-curvature conditions [25,26].

**Figure 10:**
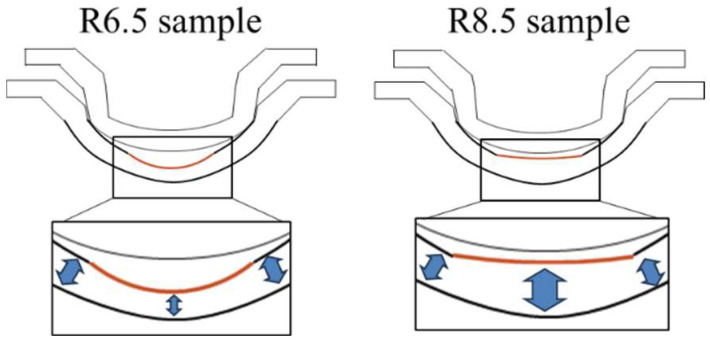
The image of the resin mold shape depending on the dry lens design.

It has been reported that in injection-molded optical lenses, geometric errors caused by asymmetric shrinkage or warpage manifest as low-order Zernike aberrations, such as astigmatism and coma, and that these errors exert a greater impact on optical performance as the curvature increases [20]. In this study, comparing the two designs confirmed that the 6.5 mm design, which has higher curvature, exhibited larger fluctuations in both astigmatism and coma, consistent with previous findings. This behavior is attributed to the structural characteristic of the 6.5 mm design, where the resin mold thickness is thinnest in the OZ region (Figure 10). This unbalanced structure likely increased the discrepancy in dry lens power and affected optical performance.

Generally, regions with thinner resin mold thicknesses are prone to non-uniform cooling rates and concentrated residual stresses, which manifest as morphological variations [21,27]. Especially under small OZ radius of curvature conditions, these geometric errors stemming from local thickness variations amplify the dry lens power and low-order aberrations. Consequently, this had a substantial impact on the final optical performance, including the deviation from the design power.

Conversely, the 8.5 mm design, with its smaller curvature, exhibited lower sensitivity to geometric variations. The impact of minute geometric errors on the power remained limited, with the fluctuation range confined to approximately ±0.2 D. Therefore, the shape-transfer from the resin mold to the dry lens was performed relatively stably. This trend aligns with reports of higher molding stability in lower-curvature conditions [27,28]. Regarding the resin mold thickness, the 8.5 mm design features a relatively thicker OZ region. Although thickness differences relative to the peripheral areas still exist, the impact of geometric variations caused by the resin mold thickness is considered smaller than that in the 6.5 mm design.

### 5.3. Importance of Resin Mold Thickness Design and Optimization Guidelines

The results from Section 5.2 yield the critical insight that variations in wavefront aberration observed in the resin mold are not necessarily transferred directly as power fluctuations in the dry lens. This phenomenon is likely due to micro-scale surface irregularities and high-order aberration components being relaxed or averaged out by flow and polymerization shrinkage as the monomer transitions from a liquid to a solid state [26]. Consequently, the dry lens power is suggested to be more strongly governed by macro-scale curvature variations and internal stress distributions arising from the resin mold thickness, rather than the wavefront aberration of the resin mold itself [20,26].

This study shows that resin mold thickness is the dominant design factor. In thinner designs, stress relaxation during cooling and polymerization is insufficient, increasing the likelihood of asymmetric shrinkage and geometric errors. Conversely, a thicker resin mold enhances morphological stability by dispersing internal stress, thereby improving adherence to the design curvature and the reproducibility of the dry lens power. Specifically, the optimal combination identified in this study—a 0.9 mm resin mold thickness, 70 MPa holding pressure, and a slow injection speed—decreased astigmatism by 95% and reduced power deviation by more than 1.5 D in high-curvature designs.

In designs with a small OZ radius of curvature, the non-uniformity in the resin mold thickness significantly affects optical performance. Therefore, a design approach that prioritizes the thickness distribution, including the area surrounding the OZ region, is essential. However, even after parameter optimization, a residual dry lens power error (approximately +1.3 to +1.5 D) is expected to persist as a direct consequence of resin shrinkage and deformation during polymerization. Future mold designs must therefore account for this molding shrinkage, with a particular focus on the OZ radius of curvature and its peripheral geometry. These findings demonstrate that the resin mold should not be treated merely as an intermediate step but must be engineered as a high-precision component that dictates the final optical characteristics of the dry lens.

## 6. Summary

This study focuses on the injection molding process, which plays a central role in the mass production of CL, and analyzes from multiple perspectives how the design of resin molds and molding conditions influence the optical properties of the dry lens. Based on orthogonal array experiments, the effects of the OZ radius of curvature, resin thickness, holding pressure, and injection speed on wavefront aberration and refractive power were comprehensively evaluated using a multi-faceted approach that included reflective wavefront sensing, OCT, and NIMO. The results revealed that resin thickness is the dominant factor in suppressing astigmatism aberration; increasing thickness from 0.7 mm to 0.9 mm reduced the aberration by approximately 95%. Furthermore, a high holding pressure of 70 MPa and a low injection speed of 5 mm/s were found to be effective in reducing coma aberration. However, a specific challenge emerged for designs with a small radius of curvature, where minute shape errors are amplified into refractive power errors, leading to a deviation of up to approximately +2.8 D from the design value, which is highly sensitive to both lens geometry and molding parameters. In contrast, for designs with low curvature, the sensitivity to molding factors was low, confirming that highly stable shape transfer within ±0.2 D is possible. As a conclusion of this study, we propose that a two-step approach is an extremely effective guideline for achieving high-precision lens manufacturing: prioritizing appropriate resin mold thickness design and optimizing molding conditions, while simultaneously incorporating compensatory designs that account for molding and polymerization shrinkage in high-curvature geometries. The implementation of real-time aberration monitoring systems is expected to be a highly promising advancement for the development of more complex contact lens designs. By utilizing multi-order diffractive optical elements [29], additional aberrations beyond those identified in this study may arise, and such systems would enable their accurate detection and control. The integration of these advanced sensing technologies is therefore considered an important future research objective.

## Figures and Tables

**Table 1:** Control factors used in this study.

No	Factor	Level 1	Level 2	Level 3
A	Radius of curvature [mm]	6.5	8.5	-
B	Resin mold thickness [mm]	0.7	0.8	0.9
C	Holding pressure [MPa]	50	60	70
D	Injection speed [mm/s]	5	10	15

**Table 2:** L18 orthogonal array.

Run	A	B	C	D
1	6.5	0.7	50	5
2	6.5	0.7	60	10
3	6.5	0.7	70	15
4	6.5	0.8	50	5
5	6.5	0.8	60	10
6	6.5	0.8	70	15
7	6.5	0.9	50	10
8	6.5	0.9	60	15
9	6.5	0.9	70	5
10	8.5	0.7	50	15
11	8.5	0.7	60	5
12	8.5	0.7	70	10
13	8.5	0.8	50	10
14	8.5	0.8	60	15
15	8.5	0.8	70	5
16	8.5	0.9	50	15
17	8.5	0.9	60	5
18	8.5	0.9	70	10

## Data Availability

Data are contained within the article.
